# Equity, diversity, and inclusion in post-secondary student mental health and well-being research: A scoping review protocol

**DOI:** 10.1371/journal.pone.0349684

**Published:** 2026-05-29

**Authors:** Sandy Rao, Ayomide Olayinka, Rya Buckley, Lexi Ewing, Bilal Qureshi, Thanida Kamchokchai, Khushi Patel, Kristin Cleverley

**Affiliations:** 1 University of Toronto, Toronto, Ontario, Canada; 2 Inlight Student Mental Health Research, University of Toronto, Toronto, Ontario, Canada; 3 Lawrence Bloomberg Faculty of Nursing, Toronto, Ontario, Canada; 4 Centre for Addiction and Mental Health, Toronto, Ontario, Canada; 5 Temerty Faculty of Medicine, Department of Psychiatry, University of Toronto, Toronto, Ontario, Canada; University of Florida, UNITED STATES OF AMERICA

## Abstract

**Objective:**

The objective of this scoping review is to systematically identify and map the extent, range, and nature of research that explicitly articulates Equity, Diversity, and Inclusion (EDI) within post-secondary student (PSS) mental health and well-being research, and to describe how EDI is conceptualized, operationalized, and implemented across study designs and research processes.

**Introduction:**

Research addressing PSS mental health and well-being has grown substantially; concurrently, EDI has become a prominent institutional and research priority within post-secondary systems. Despite this convergence, no evidence synthesis has systematically examined how EDI is explicitly integrated into PSS mental health and well-being research.

**Inclusion criteria:**

This review will include research studies of any design that explicitly articulate EDI within PSS mental health and well-being research. Eligible studies must focus on students enrolled in accredited post-secondary institutions and examine mental health or well-being within post-secondary education contexts.

**Methods:**

The review will be conducted in accordance with JBI methodology for scoping reviews and guided by the Population–Concept–Context framework. A three-step search strategy will be implemented. Electronic database searches will be conducted in MEDLINE, PsycINFO, CINAHL, EMBASE, ERIC, and Scopus or Web of Science, supplemented by targeted searches of eligible grey literature and reference list screening. Records will undergo title and abstract screening by two independent reviewers, followed by full-text screening. Data will be extracted using a structured, pilot-tested tool designed to capture how EDI is defined, framed, and integrated across stages of the research process. Extracted data will be synthesized using descriptive summary statistics, basic qualitative content analysis, and tabular and graphical mapping to address the review questions. The protocol was developed through sustained engagement with PSS with lived experience of mental health and well-being, including students from equity-deserving groups.

## Introduction

Post-secondary education represents a formative period in students’ academic, social, and personal development [1–3]. For many students, this stage coincides with emerging adulthood, a life stage characterized by increased independence, shifting identities, financial pressures, academic expectations, and new social environments. Experiences during this period shape educational trajectories, professional pathways, and long-term well-being [4]. Because students’ ability to navigate these experiences is strongly influenced by their mental health, mental health and well-being are therefore central to students’ capacity to engage, learn, participate, and thrive within post-secondary environments [5].

Research on post-secondary student (PSS) mental health and well-being has expanded substantially in response to growing recognition of its importance [3,6–10]. Across disciplines including psychology, public health, education, nursing, and the social sciences, researchers examine a wide range of topics related to student distress, resilience, service use, belonging, accessibility, and institutional supports. This body of research informs campus programming, service models, institutional planning, and broader policy discussions concerning student success and equity [11–14].

Students in post-secondary systems reflect diverse social identities, lived experiences, and structural positions. Within this context, Equity, Diversity, and Inclusion (EDI) have become prominent organizing principles in higher education governance, articulated through institutional policies, strategic frameworks, and sector-wide charters [11,15,16]. These commitments are embedded within strategic plans, research policies, and sector-level frameworks, including the National Standard for Mental Health and Well-being for Post-Secondary Students [5], The University Mental Health Charter Framework [17] and the Okanagan Charter [18]. Within this context, EDI forms part of the institutional landscape in which PSS mental health and well-being research is conducted.

For researchers working in PSS mental health and well-being, EDI represents an important consideration in shaping research design, participant recruitment, engagement practices, analytic approaches, and interpretation of findings. EDI may inform how populations are defined, how diversity within samples is described, how data are analyzed, and how implications are framed. As PSS mental health and well-being research continues to develop, clarity regarding how EDI is conceptualized and operationalized within research practice supports methodological transparency and shared learning across the field. A preliminary search of MEDLINE, the Cochrane Database of Systematic Reviews, and JBI Evidence Synthesis identified no existing or ongoing scoping or systematic reviews that examine how EDI is operationalized and implemented within PSS mental health and well-being. This absence indicates a lack of consolidated synthesis describing how EDI is currently applied in this field. Scoping review methodology is well suited to addressing this gap as it enables systematic mapping of heterogeneous and conceptually diverse bodies of literature, clarifies how key concepts are used in practice, and identifies patterns, inconsistencies, and gaps in existing research [19–21]. The objective of this scoping review is to map how EDI is operationalized and implemented within PSS mental health and well-being. By synthesizing evidence from studies that explicitly articulate EDI, this review aims to provide a clear overview of current research practices and to inform future methodological guidance in the PSS mental health and well-being field.

### Review question

The Population, Concept and Context (PCC) framework [20,21] is used for developing the research objective, and question(s) will also inform inclusion and exclusion criteria and, consequently, the literature search strategy.

### Primary question

1. How is EDI operationalized and implemented in PSS mental health and well-being research?

### Subset questions

1 What research designs, methodological approaches, and analytic strategies are used in studies that explicitly articulate EDI in PSS mental health and well-being research?
2 How is EDI operationalized within research practices, including participant recruitment, engagement approaches, ethical considerations, data collection, analysis, and reporting?
3 What patterns and variations are observed in the application of EDI across PSS mental health and well-being research?

## Inclusion criteria

### Participants

This review will include studies in which the primary participants are post-secondary students enrolled in formal education programs. Eligible populations include undergraduate, graduate and professional students registered at universities, colleges, polytechnics and other accredited post-secondary institutions, regardless of program of study or mode of delivery (for example, in-person, online or hybrid). Studies will be included if they examine students who are currently enrolled, on academic leave or recently enrolled where the focus of the research is explicitly linked to their experiences within post-secondary education. Consistent with contemporary research on PSS mental health and well-being, no age limits will be imposed, as students may enter or complete post-secondary programs at differing ages across regions and sociocultural contexts [8,22].

Studies that focus exclusively on staff, faculty, administrators, clinicians or other personnel will be excluded unless they are examined specifically in relation to their roles in shaping or responding to PSS mental health and well-being and include student participants or student perspectives as a central component of the research. Studies conducted in primary or secondary school settings, vocational training programs not classified as post-secondary, or community settings unrelated to post-secondary education will be excluded. Research in which mental health outcomes are examined in broader youth or young adult populations without clear specification of post-secondary enrolment or context will also be excluded.

### Concept

This scoping review focuses on EDI as the concept of interest, as such, explicit articulation of EDI refers to the direct use of EDI-related terminology by study authors within the title, abstract, introduction, methods, conceptual framing, analysis, or interpretation of findings. Eligible studies must explicitly reference equity, diversity, inclusion, EDI, or closely related formulations and must use this terminology in a way that is substantively relevant to the research. This may include, for example, framing the study through EDI, identifying EDI as part of the study purpose, integrating EDI into recruitment or engagement strategies, applying EDI in analytic or interpretive approaches, or explicitly discussing EDI-related implications. Studies will not be included if they report participant diversity only descriptively, such as listing demographic characteristics, without explicitly connecting these to EDI in the research focus, design, conduct, analysis, or interpretation. Similarly, studies that mention equity or inclusion only in a general or incidental way, without demonstrating substantive integration into the study, will be excluded. This operational definition is intended to support transparent and reproducible screening and to ensure consistency in identifying studies that explicitly engage with EDI within PSS mental health and well-being research.

### Context

This review will include studies that focus on mental health or well-being among students enrolled in post-secondary education [23]. Eligible studies must be explicitly framed as PSS mental health or well-being research and situated within post-secondary education contexts, including universities, colleges, polytechnics, and other accredited institutions offering education beyond the secondary level [23]. Studies conducted across a range of post-secondary–related settings will be included, such as academic programs, learning environments, student services, campus-based initiatives, and institutionally affiliated activities. Studies conducted outside formal post-secondary settings (e.g., community-based, online, or placement settings) will be included when the study population and research focus are explicitly identified as post-secondary students and clearly linked to post-secondary education. Studies will be excluded when this explicit post-secondary student framing is not articulated. Studies from all geographical regions will be included. No date restrictions will be applied. Only studies published in English will be included.

### Types of sources

#### Inclusions.

This scoping review will include research studies that explicitly articulate EDI, as mentioned in greater detail in the concept section, within PSS mental health and well-being research. Eligible studies may employ qualitative, quantitative, mixed-methods, or multimethod designs and must demonstrate that EDI is substantively integrated into the research focus, design, conduct, analysis, or interpretation of findings. Methodological orientation and use of participatory approaches will not be used as inclusion or exclusion criteria. Policy documents, institutional reports, opinion pieces, and editorials will be excluded.

### Exclusions

Studies will be excluded when they do not meet the inclusion criteria related to population, concept, context, or type of source. In particular, studies will be excluded when they are not focused on post-secondary students, do not explicitly articulate EDI as part of the research, or do not constitute research studies.

### Methods

The methodological framework proposed by Arksey and O’Malley [24], refined, and expanded by JBI [25], will guide this review. The scoping review will be conducted in accordance with the JBI methodology for scoping reviews, as outlined in the 2024 edition of the JBI Manual for Evidence Synthesis [20,21]. This approach is particularly well-suited for mapping the breadth of evidence, identifying key concepts, and pinpointing gaps in the literature. The review will include the following steps: defining the research question, conducting a comprehensive search for evidence, selecting studies based on inclusion criteria, charting and extracting data, and summarizing the results in a descriptive and narrative synthesis [20,21,26]. No research ethics board approvals are required for this protocol.

The protocol has been reviewed and revised through collaborative discussions within the Inlight Student Mental Health Research (SMHR) student advisors and facilitators. To further refine the protocol and align it with diverse perspectives, feedback has been solicited through consultations with interest-holders and PSS mental health and well-being leaders. The finalized protocol has been pre-registered with the Open Science Framework at https://osf.io/9nvwd/overview project: Equity, Diversity, and Inclusion in Post-secondary Student Mental Health and Well-being Research: A Scoping Review Protocol (February 15, 2026) allowing public access and ensuring methodological transparency. The anticipated timelines for record screening are May 2026 to June 2026, the data extraction will be June 2026 to August 2026, and the results are anticipated by November 2026.

### Study design

This scoping review will be conducted in accordance with the JBI methodology for scoping reviews. The review is designed to systematically identify and map the range and characteristics of research that explicitly articulates EDI within PSS mental health and well-being. A scoping review approach is appropriate given the conceptual breadth and methodological heterogeneity of this literature, and its distribution across multiple disciplines. Scoping review methodology supports examination of how key concepts are defined, operationalized, and implemented across diverse study designs and research traditions [19,27–29].

### Search strategy

The search strategy for this scoping review will be systematic and designed to locate peer-reviewed published studies and eligible grey literature that examine EDI within PSS mental health and well-being research. The strategy will be developed and implemented in accordance with the three-step search process recommended by JBI [20,21,26,30]. The search will be structured to capture the Population, Concept and Context elements of the review and to ensure sensitivity to interdisciplinary work across health, education and social science fields.

Step 1 involved an initial limited search of MEDLINE (via Ovid) and PsycINFO (via Ovid) to identify benchmark studies that align closely with the objectives of this review. These benchmark studies were used to inform the development of the search strategy. Titles, abstracts and index terms (for example, MeSH terms in MEDLINE and APA Thesaurus terms in PsycINFO) were examined to identify frequently used keywords and controlled vocabulary related to post-secondary students, higher education contexts, mental health and EDI. Preliminary combinations of terms such as “postsecondary” or “higher education” or “university students,” together with “mental health” or “psychological distress,” and “equity” or “EDI” were tested to assess relevance and to refine the balance between sensitivity and specificity. Insights from this preliminary search, including identification of discipline-specific terminology used in education, sociology and public health, informed the construction of the full search strategies.

Step 2 will consist of a comprehensive search of multiple bibliographic databases, with the refined strategy adapted to the indexing structure and syntax of each platform. The core databases will include MEDLINE (via Ovid), PsycINFO (via ProQuest), CINAHL (via EBSCOhost), EMBASE (via Ovid), ERIC (via ProQuest), and Scopus or Web of Science to ensure broad interdisciplinary coverage across health, education, and social science literature. In each database, the search will combine controlled vocabulary (for example, MeSH terms in MEDLINE, Emtree terms in EMBASE, CINAHL Headings in CINAHL, the APA Thesaurus in PsycINFO, and ERIC descriptors) with free-text terms searched in titles and abstracts. Boolean operators (AND, OR), truncation, wildcards, and proximity operators (where available) will be used to combine terms representing the three PCC elements: post-secondary students and post-secondary education settings (for example, “postsecondary students,” “university students,” “college students,” “higher education”), mental health and well-being (for example, “mental health,” “mental wellbeing,” “student wellbeing,” “psychological wellbeing”), and Equity, Diversity, and Inclusion (for example, “equity, diversity and inclusion,” “EDI,” “equity,” “diversity,” “inclusion”).

Validated search filters or hedges for mental health and higher education, where available, will be used as starting points and adapted to the aims of this review. Filters developed by organizations such as the Canadian Agency for Drugs and Technologies in Health or the McMaster Health Knowledge Refinery Hedges Project may be consulted [31–33]. All database searches will be documented in detail, including full search strings, database platforms, dates of execution and the number of records retrieved. The search strategy will be peer reviewed by an experienced information specialist using established tools such as the Peer Review of Electronic Search Strategies (PRESS) checklist to enhance transparency and rigour [34].

Step 3 will include supplementary search techniques to identify additional relevant evidence. The reference lists of all included studies and relevant evidence syntheses will be screened manually to identify additional sources that meet the inclusion criteria. Citation tracking of key benchmark articles will be conducted using tools such as Scopus or Web of Science to identify more recent studies that cite these works. Targeted searches will be conducted to identify unpublished or non–peer-reviewed research studies, such as graduate theses or dissertations, relevant to PSS mental health and well-being. Grey literature sources will be considered eligible only when they constitute research studies and explicitly articulate EDI within the research focus, design, analysis, or interpretation. Policy documents, institutional reports, guidance documents, and opinion pieces will not be included. The Grey Matters checklist developed by the Canadian Agency for Drugs and Technologies in Health will be used, where appropriate, to guide the identification and documentation of grey literature sources [35]. Records identified through all search methods will be imported into reference management software, deduplicated, and screened for study selection in accordance with the inclusion and exclusion criteria (S1 File).

### Search Validation

The search strategy will undergo a structured validation process to ensure accuracy and completeness. Validation will begin with iterative testing of the draft search strings in MEDLINE (via Ovid), which serves as the primary database for benchmark comparison. Retrieved records will be examined to confirm that known key studies are captured consistently and that irrelevant retrieval remains manageable. Adjustments to controlled vocabulary, free-text terms, Boolean logic, proximity operators and truncation will be made as needed to refine sensitivity and precision. Parallel testing in PsycINFO, CINAHL, EMBASE, ERIC and Scopus or Web of Science will be conducted to account for differences in indexing structures and database-specific search functionalities. The final search strategy will be reviewed using the PRESS guidelines. An experienced information specialist or librarian will apply the PRESS checklist to assess the search for completeness, appropriate use of indexing terms, logical structure, spelling and syntax accuracy, and alignment with the Population, Concept and Context elements of the review. Feedback from the PRESS review will be incorporated into the final versions of the search strategies for each database. All modifications made during this process will be documented to ensure transparency and reproducibility.

### Documentation and reporting

All aspects of the search strategy and evidence retrieval process will be documented to ensure transparency and reproducibility. Detailed records will be maintained for each database searched, including platform, search dates, full search strings, controlled vocabulary, free-text terms and any filters applied. The number of records retrieved from each source will be recorded, along with the number removed during deduplication. The complete search strategy for MEDLINE will be included as an appendix, and search strategies for all other databases will be provided in supplementary materials or upon request. Reference management procedures will be documented, including the software used for citation management, deduplication rules and any automated or manual processes applied during record cleaning. All records identified through database searching, grey literature searching, citation tracking and reference list screening will be logged and stored in a secure database to maintain an audit trail. The study selection process will be reported using the PRISMA Extension for Scoping Reviews (PRISMA-ScR) flow diagram [36]. This diagram will illustrate the number of records identified, screened, assessed for eligibility and included in the final synthesis, with reasons for exclusion documented at the full-text stage. Decisions made during the screening process, including conflicts resolved through consensus, will be recorded.

Screening calibration exercises will be documented to ensure consistency between reviewers. This will include the number of calibration rounds conducted, the level of agreement achieved and any refinements made to the inclusion and exclusion criteria to support reliable decision-making [26,27,29]. Procedures for documenting grey literature searches will include recording search terms, website URLs, dates accessed, the number of records screened and the rationale for inclusion or exclusion of identified documents. Use of the Grey Matters checklist will be documented to ensure consistent application across grey literature sources.

Any amendments to the protocol, including modifications to the search strategy, eligibility criteria, data extraction approach or analysis plan, will be recorded with dates and a rationale for each change. These amendments will be reported in the final publication to maintain transparency. Data extraction procedures will also be documented, including the development and piloting of the data extraction tool, modifications made during piloting, and decisions regarding how data will be charted when studies address multiple concepts or population groups [21,37,38]. All extracted data will be managed in a secure and organized system to ensure accuracy and traceability. Documentation and reporting will follow the methodological guidance of the JBI for scoping reviews and the PRISMA-ScR checklist (S1 Checklist) to ensure comprehensive and standardized reporting across all stages of the review.

### Study/Source of Evidence Selection

Following the search, all identified records will be imported into Covidence (Veritas Health Innovation, Melbourne, Australia) for reference management, de-duplication, and study selection. Covidence’s automated de-duplication function will be used to identify and remove duplicate records prior to screening. The number of records identified, duplicates removed, and records retained for screening will be documented to support transparency and reproducibility. Screening will occur in two stages: title and abstract screening followed by full-text screening. At both stages, studies will be assessed against the predefined inclusion criteria.

### Screening Process

Screening will occur in two stages: title and abstract screening followed by full-text screening. All screening processes will be managed using Covidence (Veritas Health Innovation, Melbourne, Australia). Title and abstract screening will be conducted independently by two reviewers using the predefined inclusion criteria. Prior to formal screening, a pilot screening exercise involving approximately 20–30 records will be undertaken to support consistent interpretation and application of the eligibility criteria. Pilot results will be reviewed collaboratively, and screening guidance refined as needed to promote alignment between reviewers. During title and abstract screening, each reviewer will classify records as either potentially relevant or exclude. Records marked as potentially relevant by at least one reviewer will automatically advance to full-text screening in Covidence.

Records marked as exclude by both reviewers will be removed from further consideration. Interrater reliability during title and abstract screening will be assessed using Cohen’s kappa coefficient, calculated within Covidence. Agreement will be examined following the pilot screening phase and upon completion of title and abstract screening. If interrater agreement is lower than anticipated, reviewers will pause screening to review discrepancies, clarify eligibility criteria, and refine screening guidance before proceeding. Any refinements will be documented to ensure transparency.

Full-text screening will be conducted by a primary reviewer with subject-matter expertise in EDI and post-secondary mental health research. The review team was intentionally structured to include this expertise, given the emerging nature of the evidence base and the absence of prior scoping reviews in this area. The inclusion criteria require careful and consistent interpretation of how EDI is explicitly articulated and substantively integrated within studies, including attention to conceptual nuance and terminology. This approach supports rigour and sensitivity in applying the criteria while maintaining clearly defined procedures to ensure reproducibility. To support methodological rigour, a second reviewer will independently verify a subset of full-text articles, with a target of achieving at least 90% agreement [39], and will be consulted when uncertainty arises regarding study eligibility. If disagreement persists, a third reviewer will provide an independent assessment. The Principal Investigator will make final decisions when required, with rationale documented. Prior to screening, reviewers will complete a pilot calibration exercise to standardize application of the inclusion and exclusion criteria. All inclusion and exclusion decisions, including reasons for exclusion at the full-text stage, will be systematically documented to maintain a transparent and auditable record of the screening process.

The study selection process will be reported in the final scoping review using a PRISMA-ScR flow diagram, detailing the number of records identified, screened, included, and excluded at each stage of the review [36], detailing the number of records identified, screened, included and excluded at each stage of the review.

### Data extraction

Data extraction will be conducted using a structured, pilot-tested extraction tool developed in alignment with the review objectives and JBI guidance for scoping reviews [26,27,29,37,38]. The tool is designed to systematically capture how EDI is operationalized and implemented within post-secondary student mental health and well-being research across diverse study designs and contexts. To support transparency and consistency, EDI-related constructs will be categorized across three interrelated domains: EDI conceptualization, EDI integration across research stages, and EDI-relevant outputs. EDI conceptualization will include the explicit terminology used by authors, definitions where provided, framing language, and the stated rationale for including EDI. EDI integration will capture whether and how EDI is reflected in the research focus, design, recruitment and sampling, engagement approaches, ethics considerations, data collection, analysis, interpretation, and reporting. EDI-relevant outputs will include key findings, implications or recommendations, and author-identified gaps related to EDI. Additional data extracted will include study identification details, population characteristics as reported by authors, post-secondary context, and methodological features (S2 File).

The extraction tool will be piloted on a sample of five to ten included studies and refined as needed, with all modifications documented. Data extraction will be conducted by one reviewer and independently verified by a second reviewer to ensure consistency, with a target of achieving at least 90% agreement [39]. The same reviewer will be consulted when uncertainty arises, with disagreements resolved through discussion or, if needed, adjudication by a third reviewer. Final decisions will be made by the Principal Investigator when required, with rationale documented.

The finalized extraction tool will be included as an appendix in the final review. Consistent with scoping review methodology, no critical appraisal of individual sources will be undertaken, as the purpose of this review is to map the extent, range, and nature of evidence describing the use of EDI within PSS mental health and well-being research [26,27,29,38].

### Data analysis and presentation

Data analysis will involve descriptive synthesis, basic qualitative content analysis and mapping of extracted data to address the review questions [40]. Descriptive analytical methods will be used to summarize study characteristics, including population features, geographic location, post-secondary context, methodological approaches, and how EDI is articulated and integrated within PSS mental health and well-being research. Extracted data will be categorized to describe patterns in how EDI is conceptualized, operationalized, and implemented across studies, including variation in definitions, research design choices, methodological strategies, and reporting practices. The qualitative content analysis will focus on identifying patterns, similarities, and variations in the application of EDI across the literature, as well as areas where evidence is limited or unevenly developed [40]. Findings will be mapped descriptively rather than interpreted through predefined theoretical or justice frameworks, consistent with the scoping review objective of clarifying current research practices.

Results will be presented using tables, figures, and narrative synthesis. Tabular summaries will describe key study characteristics and how EDI is integrated across stages of the research process. Graphical displays (e.g., frequency tables or charts) will be used to illustrate the distribution of study characteristics and analytic patterns. A narrative synthesis will accompany the tables and figures to integrate findings across categories and explicitly link results to the review questions. Findings will be presented in a manner intended to support interpretability for multiple audiences, including students, researchers, and other knowledge users, with emphasis on clarity, transparency, and relevance to future methodological development in PSS mental health and well-being research [20,27,30,38].

### Patient and Public Involvement

This scoping review has been informed by sustained collaboration with post-secondary students who bring lived experience of mental health and well-being within institutional contexts. Four students from different departments and faculties, representing equity-deserving groups, are engaged as knowledge partners in the review. These students serve as facilitators and leaders within the Inlight SMHR at the University of Toronto, where student engagement and leadership are central to research development and knowledge mobilization. The conceptual foundations of this review build upon prior student-led EDI work conducted within Inlight SMHR. Student partners contributed to shaping the focus of the review, refining the research questions, and informing the conceptual framing of how EDI is understood within PSS mental health and well-being research. Their perspectives have guided decisions related to scope, terminology, and interpretive orientation. Post-secondary students will continue to be engaged throughout the review process, including participation in interpretive discussions, refinement of analytic categories, and consideration of implications for research practice. They will also contribute to knowledge mobilization to support accessibility and relevance for student and institutional audiences. This approach aligns with current guidance on meaningful interest-holder involvement in scoping reviews by integrating student leadership and lived experience into the design and interpretation of the review [21,28,29,38,41].

### Knowledge Mobilization

Knowledge mobilization for this scoping review is embedded within an established and collaborative knowledge exchange infrastructure housed within Inlight SMHR. The approach reflects the principles of reciprocity, shared ownership and ongoing engagement that underpin the broader organization. The strategy draws on existing mechanisms for communication, dissemination and exchange that are routinely used within the lab, including monthly newsletters, public talks, learning engagements, the national research network, research fellows and the student advisory council. These established structures support a sustained and multidirectional flow of knowledge between researchers, students, community partners and policy actors [42,43]. The student advisory council at Inlight SMHR plays a key role in embedding reciprocity in the knowledge mobilization process. Members will be invited to comment on emerging themes, reflect on their relevance to institutional conditions and identify areas requiring deeper attention or more accessible dissemination. This approach ensures that findings are mobilized in a manner that is meaningful to students, who are directly affected by PSS mental health and well-being research, policies and practices. Their contributions will help shape communication materials, interpretive summaries and recommendations.

## Supporting information

S1 Checklist PRISMA-ScR checklist. (DOCX)

S1 File Search Strategy. (DOCX)

S2 File Data Extraction. (DOCX)
