# Supplementary material for: Equity, diversity, and inclusion in post-secondary student mental health and well-being research: A scoping review protocol
Source: PLoS One. 2026 May 29;21(5):e0349684. doi: 10.1371/journal.pone.0349684 (PMC13221019; doi:10.1371/journal.pone.0349684)
Supplement: S1 File — (DOCX) [file pone.0349684.s002.docx]

### Search strategy

**Database:**
Ovid MEDLINE(R) ALL <1946 to February 04, 2026>

| **#** | **Query** | **Results from 5 Feb 2026** |
| --- | --- | --- |
| 1 | exp Students/ | 196,671 |
| 2 | "student*".kf,tw. | 435,184 |
| 3 | 1 or 2 | 473,075 |
| 4 | exp Universities/ | 61,603 |
| 5 | (postsecondary or "post-secondary" or "higher education" or tertiary education).tw,kf. | 33,297 |
| 6 | (post-secondary or postsecondary or postgraduate* or graduate or graduates or undergrad* or under-grad* or doctora* or PHD or master or masters or academ* or cegep or tertiary).tw,kf. | 725,238 |
| 7 | (education* institution* or universit* or college* or polytechnic* or cegep).tw,kf. | 711,715 |
| 8 | 4 or 5 or 6 or 7 | 1,353,263 |
| 9 | 3 and 8 | 217,306 |
| 10 | exp Mental Health/ | 76,847 |
| 11 | exp Mental Disorders/ | 1,588,201 |
| 12 | ((mental* or psychol* or psychi*) adj2 (health or disorder* or condition* or ill or disease* or illness* or disabilit* or problem* or challeng*)).tw,kf. | 507,164 |
| 13 | ((mental* or psychol* or psychi*) adj2 (stress* or distress* or well or wellness or wellbeing or well-being)).tw,kf. | 104,203 |
| 14 | 10 or 11 or 12 or 13 | 1,942,951 |
| 15 | 9 and 14 | 35,858 |
| 16 | exp Diversity, Equity, Inclusion/ | 469 |
| 17 | ("equity diversity inclusion" or "equity, diversity inclusion" or "EDI" or "diversity, equity inclusion" or "diversity equity inclusion" or "DEI").tw,kf. | 9,254 |
| 18 | (equity adj3 diversity).tw,kf. | 3,147 |
| 19 | (equity adj3 inclusion).tw,kf. | 3,244 |
| 20 | (diversity adj3 inclusion).tw,kf. | 4,333 |
| 21 | 16 or 17 or 18 or 19 or 20 | 13,588 |
| 22 | 15 and 21 | 132 |

### Concept Map

| **Population (P) Post-secondary students** | **Context (C) Mental health & well-being (research)** | **Concept (C) Explicit EDI only** |
| --- | --- | --- |
| Students/ (MeSH) | Mental Health/ (MeSH) | “equity, diversity and inclusion” |
| Education, Higher/ (MeSH) | Mental Disorders/ (MeSH) | “equity diversity inclusion” |
| Universities/ (MeSH) *(optional)* | mental health | “equity, diversity, inclusion” *(no “and”)* |
| (student* adj3 (universit* or college* or postsecondary or post-secondary or higher education or tertiary)).tw,kf. | wellbeing OR well-being OR wellness | “equity diversity and inclusion” *(variant)* |
| (higher education institution* or HEI or tertiary institution*).tw,kf. | (mental* or psychol* or psychi*) adj2 (health or disorder* or condition* or ill or disease* or illness* or problem* or challeng*).tw,kf. | “diversity, equity and inclusion” |
| (undergraduate* or graduate* or postgraduat*).tw,kf. AND (universit* or college* or higher education).tw,kf. *(optional precision line)* | (mental* or psychol* or psychi*) adj2 (stress* or distress* or well or wellness or wellbeing or well-being).tw,kf. | “diversity equity inclusion” |
|  |  | EDI OR DEI *(as acronyms)* |
|  |  | (equity adj3 divers* adj3 inclus*).tw,kf. *(optional)* |
|  |  | Health Equity/ (MeSH) *(optional; test)* |
