# Supplementary material for: Equity, diversity, and inclusion in post-secondary student mental health and well-being research: A scoping review protocol
Source: PLoS One. 2026 May 29;21(5):e0349684. doi: 10.1371/journal.pone.0349684 (PMC13221019; doi:10.1371/journal.pone.0349684)
Supplement: S2 File — (DOCX) [file pone.0349684.s003.docx]

### Data extraction instrument

The data extraction instrument for this scoping review was developed specifically for this study using Excel to align with the review’s objectives and the PCC framework. The tool is designed to ensure clarity, consistency, and ease of analysis while allowing for the automatic generation of descriptive summaries, tables, and charts to support reporting. This Excel-based tool ensures the extraction process is systematic and consistent while allowing for seamless analysis and reporting. The full finalized version of the tool is available upon request.

| **Section** | **Field** | **What to Extract** |
| --- | --- | --- |
| **A. Citation & Record Management** | Study ID | Unique ID assigned by reviewers |
|  | Authors | As cited |
|  | Year | Year of publication |
|  | Title | Full title |
|  | Source / Journal | Journal or source |
|  | Country / Region | Country or region of study |
|  | Publication Type | Peer-reviewed article / Thesis–dissertation / Other research study |
|  | DOI / URL | If available |
| **B. Eligibility Confirmation** | Post-secondary students included? | Yes/No + short descriptor |
|  | PSS mental health and well-being focus present? | Yes/No + phenomenon (1–3 words) |
|  | Explicit EDI terminology used | Verbatim term(s): e.g., EDI, equity, inclusion, DEI |
|  | EDI substantively integrated | Yes/No + 1-line justification |
| **C. Study Characteristics** | Aim / Purpose | 1–2 sentences maximum |
|  | Study Design | Qualitative / Quantitative / Mixed-methods / Multimethod |
|  | Methods / Data Sources | Survey, interviews, focus groups, administrative data, document analysis, other |
|  | Analytic Approach | Thematic/qualitative, statistical, mixed integration, other (as stated) |
| **D. Population & Post-secondary Context** | Student Level | Undergraduate / Graduate / Professional / Mixed / Not stated |
|  | Institution Type | University / College / Polytechnic / Multiple / Not stated |
|  | Recruitment / Sampling Channel | Short phrase (e.g., email list, service users, class-based) |
|  | Equity-relevant Characteristics Reported | Tick all reported: race/ethnicity, Indigeneity, gender, disability, SES, international status, sexual orientation, other |
| **E. EDI Conceptualization** | Definition of EDI | Verbatim sentence or tight paraphrase (if provided) |
|  | EDI Framing Language | Equity, diversity, inclusion, justice, anti-racism, intersectionality, decolonizing, accessibility, equity-deserving, other |
|  | Rationale for Including EDI | 1 sentence |
| **F. EDI Integration Across Research Stages** | Research focus shaped by EDI | Yes/No + short note |
|  | Design shaped by EDI | Yes/No + short note |
|  | Recruitment/sampling reflects EDI | Yes/No + short note |
|  | Engagement approach reflects EDI | Yes/No + short note |
|  | Ethics considerations reflect EDI | Yes/No + short note |
|  | Data collection reflects EDI | Yes/No + short note |
|  | Analysis reflects EDI | Yes/No + short note |
|  | Interpretation reflects EDI | Yes/No + short note |
|  | Reporting practices reflect EDI | Yes/No + short note |
| **G. EDI-Relevant Outputs** | Key EDI-related Findings | Up to 3 bullet points (EDI only) |
|  | Implications / Recommendations | Up to 3 bullets (research-focused) |
|  | Author-identified EDI Gaps | 1–2 bullets |
